# Relationship among Phosphorus Circulation Activity, Bacterial Biomass, pH, and Mineral Concentration in Agricultural Soil

**DOI:** 10.3390/microorganisms5040079

**Published:** 2017-12-04

**Authors:** Dinesh Adhikari, Tianyi Jiang, Taiki Kawagoe, Takamitsu Kai, Kenzo Kubota, Kiwako S. Araki, Motoki Kubo

**Affiliations:** Department of Biotechnology, Faculty of Life Science, Ritsumeikan University, Nojihigashi 1-1-1, Kusatsu, Shiga 525-8577, Japan; dinesh@sofixagri.com (D.A.); jerrylittlebro@gmail.com (T.J.); gr0320rp@ed.ritsumei.ac.jp (T.Kaw); kai_takamitsu@meiji.ac.jp (T.Kai); kenzo-k@daiwahouse.jp (K.K.); kiwakosa@fc.ritsumei.ac.jp (K.S.A.)

**Keywords:** phosphorus circulation activity, bacterial biomass, soil pH, mineral concentration, agricultural soil

## Abstract

Improvement of phosphorus circulation in the soil is necessary to enhance phosphorus availability to plants. Phosphorus circulation activity is an index of soil’s ability to supply soluble phosphorus from organic phosphorus in the soil solution. To understand the relationship among phosphorus circulation activity; bacterial biomass; pH; and Fe, Al, and Ca concentrations (described as mineral concentration in this paper) in agricultural soil, 232 soil samples from various agricultural fields were collected and analyzed. A weak relationship between phosphorus circulation activity and bacterial biomass was observed in all soil samples (*R*^2^ = 0.25), and this relationship became significantly stronger at near-neutral pH (6.0–7.3; *R*^2^ = 0.67). No relationship between phosphorus circulation activity and bacterial biomass was observed at acidic (pH < 6.0) or alkaline (pH > 7.3) pH. A negative correlation between Fe and Al concentrations and phosphorus circulation activity was observed at acidic pH (*R*^2^ = 0.72 and 0.73, respectively), as well as for Ca at alkaline pH (*R*^2^ = 0.64). Therefore, bacterial biomass, pH, and mineral concentration should be considered together for activation of phosphorus circulation activity in the soil. A relationship model was proposed based on the effects of bacterial biomass and mineral concentration on phosphorus circulation activity. The suitable conditions of bacterial biomass, pH, and mineral concentration for phosphorus circulation activity could be estimated from the relationship model.

## 1. Introduction

Phosphorus is an important nutrient element for plants that exists in various organic and inorganic forms, and phytate is a major form of organic phosphorus in soil [1,2]. The inorganic forms include soluble phosphates (such as H_2_PO_4_^−^ and HPO_4_^2−^) and insoluble compounds (such as Fe_3_(PO_4_)_2_, AlPO_4_, and Ca_3_(PO_4_)_2_). Soluble phosphates are available to plants, but their availability is regulated by several factors including microorganisms; sorption intensity over soil minerals; and precipitation with Ca, Fe, and Al in the soil.

Soil microorganisms mineralize organic phosphorus (such as phytate) into inorganic phosphates [3,4,5]. However, high concentrations of Fe, Al, and Ca (described as ‘mineral concentration’ in this paper) could limit the availability of phosphorus, even at high level microbial biomass. Soluble phosphates precipitate as Fe-, Al-, and Ca-phosphates in the soil at specific pH [6]. Phosphorus adsorption varies with the solubility of Fe, Al, or Ca at acidic or alkaline pH [7,8].

Understanding the phosphorus circulation process in soil is important as most of the agricultural soils contain a little amount of available phosphorus [9]. Phosphorus availability has generally been considered to be controlled by physical and chemical processes such as sorption–desorption and pH driven precipitation-dissolution. However, biological processes such as mineralization of organic phosphorus can also have a considerable role on phosphorus availability [10,11]. The role of such biological processes is more important where plant available phosphorus is generally low and the fraction of organic phosphorus is higher than the inorganic forms [12,13].

Improvement of phosphorus circulation in the soil is necessary to enhance phosphorus availability to plants. Although the effects of microorganisms, pH, and minerals (such as Fe, Al, and Ca) on phosphorus availability have been independently investigated [3,4,5,6,7,8], the total effects of these factors on phosphorus circulation activity in the soil are still unclear. In this study, the relationship among phosphorus circulation activity, bacterial biomass, pH, and mineral concentration (Fe, Al, and Ca) were analyzed. In addition, a relationship model was constructed based on the results.

## 2. Materials and Methods

### 2.1. Soil Sampling and Preparation

The soil samples used in this study were collected from 232 agricultural fields in Japan, Afghanistan, and France. Samples of Japan and Afghanistan belong to various upland annual crop fields. The samples from France correspond to the orchard fields. The general climates of Japan, France, and Afghanistan are temperate, temperate, and sub-tropical, respectively. The samples were taken from a depth of about 15 cm after removing the surface crust or litter layer and sieved through 2 mm mesh size stainless steel screens before analysis. The soil samples were transported to the laboratory located in Ritsumeikan University, Shiga, Kusatsu, Japan, soon after sampling without drying and kept cool at 4 °C until the analysis. The samples from Japan were transported to the laboratory within one week and those from France and Afghanistan were transported within four weeks after sampling.

### 2.2. Determination of Soil pH and Metal Concentration (Fe, Al, and Ca)

Soil pH (1:2.5 soil-to-water suspension, *w*/*v*) was analyzed using a pH meter (LAQUA F-72, Horiba, Kyoto, Japan). The diethylenetriaminepentaacetic acid (DTPA) method was used to extract the Fe concentration from soil [14]. Similarly, Ca was extracted by the ammonium acetate method [15], while Fe and Ca concentrations in the extracts were measured using an atomic absorption spectrophotometer (Hitachi Z2300, Hitachi High-Technologies Corporation, Tokyo, Japan). Finally, the Al concentration in soil was estimated using a titrimetric method after extracting the soil sample with KCl [16]. The forms of Fe, Ca, and Al were named “available Fe”, “exchangeable Al”, and “exchangeable Ca” according to the previous reports [15,17,18].

### 2.3. Analysis of Bacterial Biomass and Phosphorus Circulation Activity

Soil bacterial biomass was analyzed by the slow-stirring method [19]. The phosphorus circulation activity was evaluated as previously described [20]. The phosphorus circulation activity indicates the rate of increase in soluble phosphorus in soil after addition of an organic phosphorus substrate (phytic acid). The method of determining phosphorus circulation activity is summarized as follows:

- One gram soil was placed in four centrifuge tubes.
- A measure of 150 μL of phytic acid solution (containing 3.3 mg organic phosphorus) was added in two tubes (Tube P), and 150 μL of distilled water was added in the remaining two tubes (Tube W).
- In each of the phytic acid added tubes (Tube P0) and distilled water-added tubes (Tube W0), the water extractable phosphorus was analyzed immediately after the addition of water or phytic acid solution by molybdenum blue method [21].
- The remaining phytic acid added and distilled water added tubes (Tubes P3 and W3, respectively) were incubated at 25 °C for three days and analyzed for the water-extractable phosphorus.
- The phosphorus circulation activity was calculated by using the following formula:
  $$Phosphorus\;circulation\;activity\;\left(point\right)=\;\frac{\left(\mathrm{Soluble}\;P\;\mathrm{in}\;\mathrm{Tube}\;P3-\mathrm{Soluble}\;P\;\mathrm{in}\;\mathrm{Tube}\;P0\right)-\left(\mathrm{Soluble}\;P\;\mathrm{in}\;\mathrm{Tube}\;W3-\mathrm{Soluble}\;P\;\mathrm{in}\;\mathrm{Tube}\;W0\right)}{\mathrm{Added}\;P\;\mathrm{in}\;\mathrm{Tube}\;P}\times 100$$

**Figure 1:**
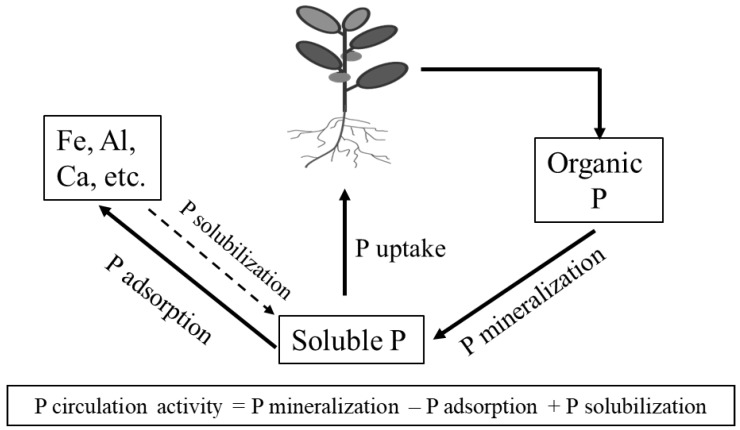
Schematic diagram showing the phosphorus circulation activity in soil.

A schematic diagram showing the phosphorus circulation activity used in this study is shown in Figure 1. The phosphorus circulation activity is increased with mineralization and solubilization activities and decreased with adsorption. This method considers the solubilization factor constant in both phytate-added and water-added samples. The phosphorus circulation activity was expressed by assigning 0 points for no mineralization or complete adsorption to 100 points for complete mineralization but no adsorption.

To define the categories of bacterial biomass level for high, medium, and low level of phosphorus circulation activities, a reference from our previous study was taken [22]. In that study, more than 75% of the soil samples showing high (66.6–100.0 points) or low (<33.3 points) levels of phosphorus circulation activity had higher (≥6.0 × 10^8^ cells/g) or lower (<4.3 × 10^8^ cells/g) bacterial biomass (data not shown). Therefore, three categories of bacterial biomass level were defined as follows: high (≥6.0 × 10^8^ cells/g), medium (4.3 × 10^8^–5.9 × 10^8^ cells/g), and low (<4.3 × 10^8^ cells/g), respectively.

### 2.4. Determination of Total Carbon (TC), Total Nitrogen (TN), and Total Phosphorus (TP)

Total carbon in soil was estimated using a Total Organic Carbon Analyzer (TOC-VCPH, Shimadzu, Kyoto, Japan) and solid sample combustion unit (SSM-5000A, Shimadzu, Kyoto, Japan) according to the manufacturer’s instructions. Total nitrogen and TP in soil samples were extracted by Kjeldahl digestion [23]. Total nitrogen in the extract was determined by the indophenol blue method [24] and total phosphorus by the molybdenum blue method [21].

### 2.5. Preparation of Soil Samples with Identical Physico-Chemical Properties but Different Bacterial Biomasses

To examine the effect of bacterial biomass on phosphorus circulation activity in the same soil condition, five soil samples with identical physico-chemical properties but different bacterial biomasses were prepared. For this purpose, a soil (pH 6.6) was autoclaved two times and mixed with the same non-autoclaved soil at different ratios.

## 3. Results

### 3.1. Relationship between Phosphorus Circulation Activity and Bacterial Biomass

**Figure 2:**
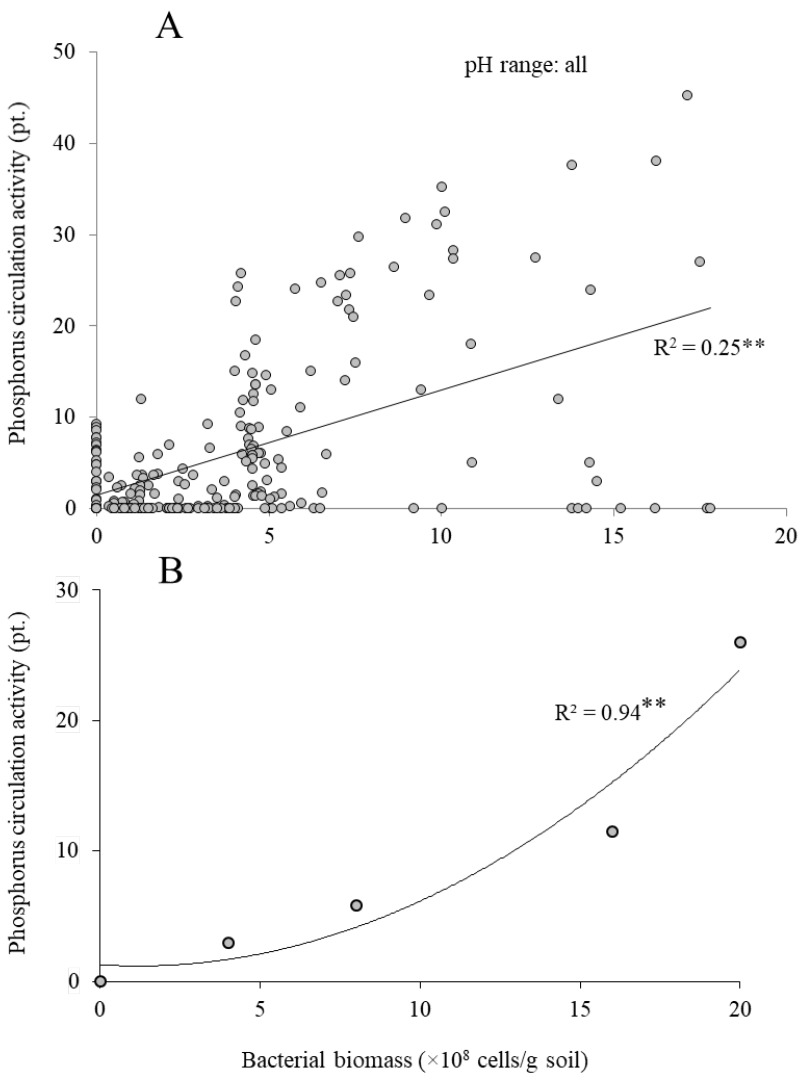
Relationship between phosphorus circulation activity and bacterial biomass in: (**A**) various agricultural soils (*n* = 232) and (**B**) the same agricultural soil with different bacterial biomass. Soils in (**B**) were prepared by mixing autoclaved soil at different ratios. *R*^2^-value marked with two asterisks (**) indicates significant regression (*p* < 0.01).

To understand the effects of bacterial biomass on phosphorus circulation activity in the soil, the relationship between the phosphorus circulation activity and bacterial biomass was analyzed. Correlation analysis of 232 various agricultural soils showed a weakly positive relationship (*R*^2^ = 0.25) (Figure 2A). When the same soil condition with different bacterial biomass was used to analyze the relationship, a significantly strong curvilinear relationship was observed (*R*^2^ = 0.94) (Figure 2B). These results suggest that not only bacterial biomass, but also other soil properties—such as pH and mineral concentration—appear to affect phosphorus circulation activity in the soil. Furthermore, the effect of bacterial biomass seems to be curvilinear.

The role of bacteria for improving phosphorus mineralization activity has been reported [25,26,27,28]. Therefore, making the soil condition suitable for bacterial growth seems to be necessary for improving phosphorus availability.

### 3.2. Effects of Soil pH and Bacterial Biomass on Phosphorus Circulation Activity

**Figure 3:**
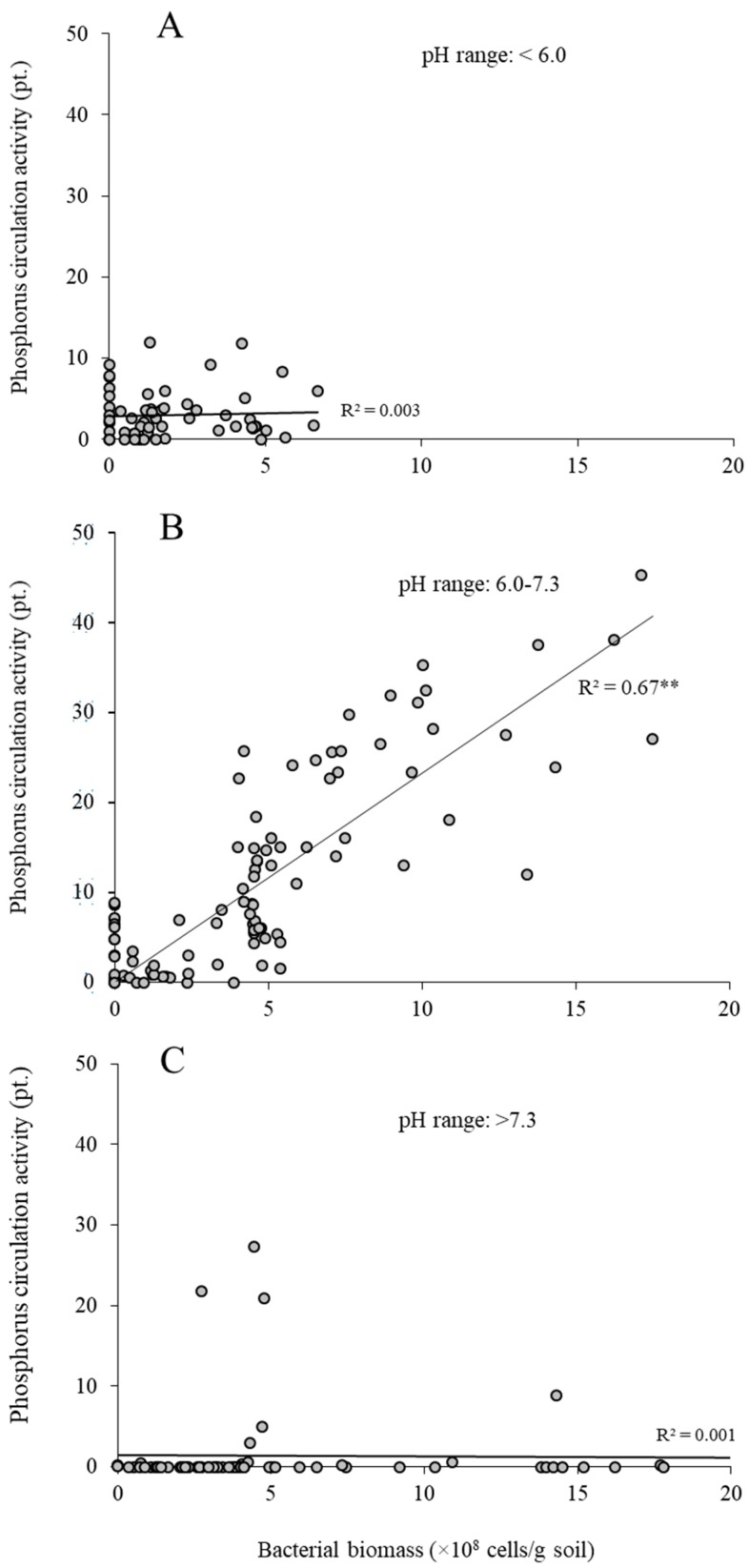
Relationship between phosphorus circulation activity and bacterial biomass in agricultural soil at different pH ranges. (**A**) Acid (pH < 6.0, *n* = 60); (**B**) near-neutral (pH 6.0–7.3, *n* = 94); (**C**) alkaline (pH > 7.3, *n* = 78). R^2^-value marked with two asterisks (**) indicates a significant regression (*p* < 0.01).

The relationships between phosphorus circulation activity and bacterial biomass under acidic (pH < 6.0), near-neutral (pH 6.0–7.3), and alkaline (pH > 7.3) soil conditions were analyzed (Figure 3A–C). A significant positive relationship at near-neutral pH range was observed (*R*^2^ = 0.67) (Figure 3B). However, no relationship was observed at acidic or alkaline pH (Figure 3A,C). The weak relationship suggests that adsorption of phosphate ions with mineral concentration (such as Fe, Al, and Ca) in the soil affects the phosphorus circulation activity [6,7,8].

### 3.3. Effects of Mineral Concentration and pH on Phosphorus Circulation Activity

**Figure 4:**
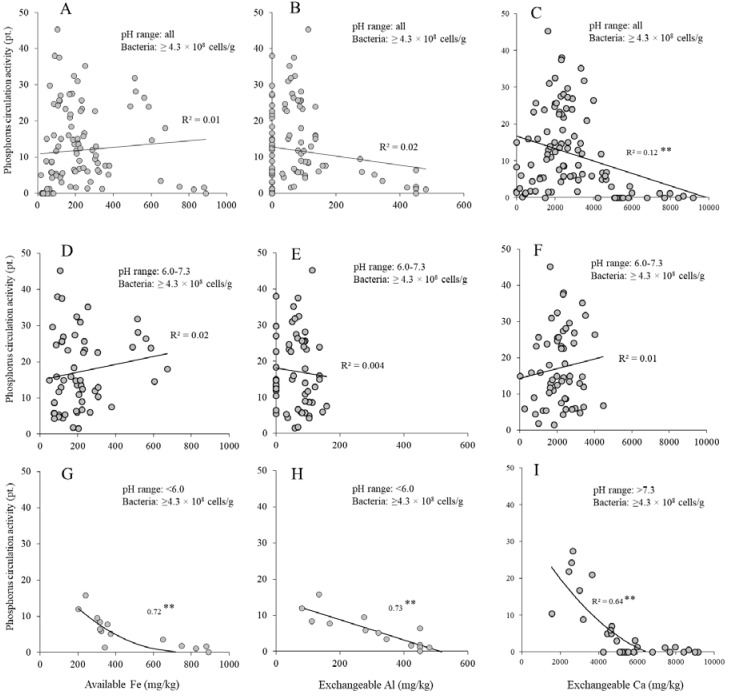
Relationship between phosphorus circulation activity and mineral concentrations at abundant bacterial biomass. (**A**) Phosphorus circulation activity and available Fe at all pH values; (**B**) phosphorus circulation activity and exchangeable Al at all pH values; (**C**) phosphorus circulation activity and available Ca at all pH values; (**D**) phosphorus circulation activity and available Fe at near-neutral pH; (**E**) phosphorus circulation activity and exchangeable Al concentration at near-neutral pH; (**F**) phosphorus circulation activity and available Ca at near-neutral pH; (**G**) phosphorus circulation activity and available Fe at acid pH; (**H**) phosphorus circulation activity and exchangeable Al at acid pH; (**I**) phosphorus circulation activity and available Ca at alkaline pH. *R*^2^-value marked by two asterisks (**) indicates a significant regression (*p* < 0.01).

To understand the relationship between phosphorus circulation activity and mineral concentration, the effects of mineral concentration (Fe, Al, and Ca) on phosphorus circulation activity at abundant bacterial biomass (≥4.3 × 10^8^ cells/g) were investigated (Figure 4A–F). No relationship between the phosphorus circulation activity and mineral concentration was observed at near-neutral pH (*R*^2^ ≤ 0.02) (Figure 4D–F). However, significant negative effects of Fe and Al concentrations at acidic pH (R^2^ values 0.72 and 0.73; Figure 4G,H, respectively) and of Ca concentration at alkaline pH (*R*^2^ value 0.64; Figure 4I) were observed. The results indicate that the effects of mineral concentration at near-neutral pH on phosphorus circulation activity were considerably low.

Fe, Al, and Ca are well known minerals that adsorb soluble phosphorus at specific pH [29,30]. No relationship at near-neutral pH suggests that adsorption of phosphate ions with minerals inhibited the phosphorus circulation activity in the soil at acidic or alkaline pH [31,32]. Therefore, factors such as bacterial biomass, pH, and mineral concentration should be considered altogether when evaluating activation of phosphorus circulation activity in the soil.

### 3.4. Suitable Soil Condition for High Phosphorus Circulation Activity

To show the relationship among phosphorus circulation activity, bacterial biomass, pH, and mineral concentration in soil, bacterial biomass, pH, and mineral concentrations were analyzed at different levels of phosphorus circulation activity (Table 1). Samples belonging to the low phosphorus circulation activity group (<5.0 points) had low bacterial biomass with either alkaline or acidic pH range. The results indicate that high bacterial biomass and low mineral concentrations lead to the enhancement of phosphorus circulation activity in soil.

Seven soils showing higher values of phosphorus circulation activity (>30 points) were selected to identify suitable soil conditions for high phosphorus circulation activity. High levels of TC, TN, and TP were observed in each soil and the average values of TC, TN, and TP were 34,300 mg/kg, 1420 mg/kg, and 3790 mg/kg, respectively (Table 2). The bacterial biomass also showed high levels (average value = 12.0 × 10^8^ cells/g), and all samples belonged to the near-neutral pH range. These results indicate that higher and balanced carbon, nitrogen, and phosphorus in soil seemed to be suitable for microbial growth and phosphorus circulation activities.

### 3.5. Relationship among Phosphorus Circulation Activity, Mineral Concentration, pH, and Bacterial Biomass

**Figure 5:**
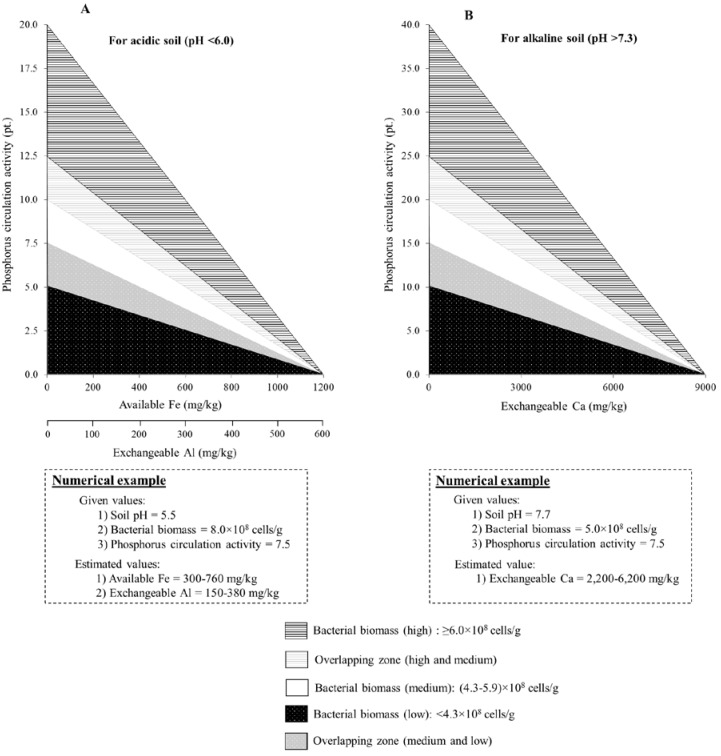
Model showing the relationship among phosphorus circulation activity, bacterial biomass, pH, and mineral concentration. (**A**) Available Fe or exchangeable Al at acidic pH range (<6.0) and (**B**) exchangeable Ca at alkaline pH range (>7.3). Examples of estimating concentrations of Fe, Al, and Ca using the model are also shown.

A model was constructed based on the positive effects of bacterial biomass and negative effects of mineral concentration (Fe, Al, and Ca) on the phosphorus circulation activity (Figure 5). The models for Fe and Al at acidic pH range and Ca at alkaline pH range were constructed separately. As shown in Figure 5, the phosphorus circulation activity, bacterial biomass, and mineral concentration were related to each other at acidic and alkaline pH. The phosphorus circulation activity varied differently in high (≥6.0 × 10^8^ cells/g), medium (4.3 × 10^8^–5.9 × 10^8^ cells/g), and low (<4.3 × 10^8^ cells/g) levels of bacterial biomass. The phosphorus circulation activity was 0 point at all categories of bacterial biomass when concentrations of Fe, Al, and Ca were ≥1200 mg/kg, ≥600 mg/kg, and ≥9000 mg/kg, respectively. Similarly, when concentrations of each mineral were low, the phosphorus circulation activity value was the highest, and the value corresponded to the bacterial biomass level. From this model, mineral concentrations can be estimated by using the values of bacterial biomass and phosphorus circulation activity. In addition, the suitable conditions of bacterial biomass, pH, and mineral concentration in soil can be estimated from this model.

Limited phosphorus availability is always a big challenge for improving agricultural productivity. Under acidic and alkaline soils, the challenge is more severe. It is reported that about 30% of the world’s ice-free land is acidic [33]. Similarly, alkaline soils occupy almost the same proportion of the world’s arable soil. Understanding the relationship among bacterial biomass, mineral concentration, pH, and phosphorus circulation activity could be a useful tool for improving phosphorus use efficiency, especially under organic farming systems.

## 4. Conclusions

The relationship between phosphorus circulation activity and bacterial biomass was significantly positive, especially at near-neutral pH in the soil, whereas the relationship between phosphorus circulation activity and concentrations of Fe, Al, and Ca was negative at acidic or alkaline pH. The relationship model of the phosphorus circulation activity, bacterial biomass, pH, and mineral concentration can be used for estimating Fe, Al, and Ca concentrations in soil using the values of phosphorus circulation activity and bacterial biomass.

## Figures and Tables

**Table 1:** Frequency distribution of samples with different levels of phosphorus circulation activity.

Phosphorus Circulation Activity (pt.)	Number of Sample	Bacterial biomass (×10^8^ cells/g)	pH	Available Fe (mg/kg)	Exchangeable Al (mg/kg)	Exchangeable Ca (mg/kg)
<1.0	89	3.4	7.5	110	110	4300
1.0–4.9	54	2.7	5.4	310	280	1270
5.0–9.9	39	3.5	6.3	230	110	2240
10.0–19.9	23	5.8	6.9	250	110	2530
20.0–29.9	20	7.0	8.1	240	80	2370
30.0–45.3	7	6.8	12.3	160	70	2390

**Table 2:** Properties of soils showing higher phosphorus circulation activity (>30 points).

Sample	Phosphorus Circulation Activity (pt.)	TC (mg/kg)	TN (mg/kg)	TP (mg/kg)	Available Fe (mg/kg)	Exchangeable Al (mg/kg)	Exchangeable Ca (mg/kg)	Bacterial Biomass (×10^8^ Cells/g)	pH
1	45	42,600	1290	3090	100	110	1610	14	6.7
2	38	45,570	1850	1910	90	80	2330	16	7.3
3	37	36,900	1390	1970	110	70	2310	13	6.7
4	35	34,270	1130	3300	250	50	3330	10	7.0
5	32	29,200	1400	2670	190	70	2000	10	6.7
6	31	23,000	1520	1720	210	60	3500	10	7.0
7	31	27,550	1350	1890	210	50	1690	10	6.5
Average	33	34,300	1420	2360	160	70	2390	12	6.8
